# PTR-MS Characterization of VOCs Associated with Commercial Aromatic Bakery Yeasts of Wine and Beer Origin

**DOI:** 10.3390/molecules21040483

**Published:** 2016-04-12

**Authors:** Vittorio Capozzi, Salim Makhoul, Eugenio Aprea, Andrea Romano, Luca Cappellin, Ana Sanchez Jimena, Giuseppe Spano, Flavia Gasperi, Matteo Scampicchio, Franco Biasioli

**Affiliations:** 1 Department of Food Quality and Nutrition, Research and Innovation Centre, Fondazione Edmund Mach (FEM), via E. Mach 1, San Michele all’Adige 38010, Italy; vittorio.capozzi@gmail.com (V.C.); salim.makhoul@fmach.it (S.M.); eugenio.aprea@fmach.it (E.A.); luca.cappellin@fmach.it (L.C.); flavia.gasperi@fmach.it (F.G.); 2 Faculty of Science and Technology, Free University of Bolzano, Bolzano 39100, Italy; andrea.romano@unibz.it (A.R.) matteo.scampicchio@unibz.it (M.S.); 3 Department of Agriculture, Food and Environment Sciences, University of Foggia, via Napoli 25, Foggia 71122, Italy; giuseppe.spano@unifg.it; 4 L’Unité Mixte de Recherche Procédés Alimentaires et Microbiologiques—L’équipe Vin Aliment Microbiologie et Stress, Institut Universitaire de la Vigne et du Vin, 1 rue Claude Ladrey, Dijon Cedex 21078, France; 5 Department of Chemistry, University of Balamand, P. O. Box 100, Tripoli, Lebanon; 6 Lallemand SAS, Lallemand Baking Solution Department, a Subsidiary of Lallemand Inc., Blagnac 31702, France; asanchez@lallemand.com

**Keywords:** bread, yeast, direct-injection mass spectrometry (DIMS), proton-transfer-reaction time-of-flight mass spectrometry (PTR-ToF-MS), volatile organic compounds (VOCs), aroma, flavour, *Saccharomyces cerevisiae*

## Abstract

In light of the increasing attention towards “green” solutions to improve food quality, the use of aromatic-enhancing microorganisms offers the advantage to be a natural and sustainable solution that did not negatively influence the list of ingredients. In this study, we characterize, for the first time, volatile organic compounds (VOCs) associated with aromatic bakery yeasts. Three commercial bakery starter cultures, respectively formulated with three *Saccharomyces cerevisiae* strains, isolated from white wine, red wine, and beer, were monitored by a proton-transfer-reaction time-of-flight mass spectrometer (PTR-ToF-MS), a direct injection analytical technique for detecting volatile organic compounds with high sensitivity (VOCs). Two ethanol-related peaks (*m*/*z* 65.059 and 75.080) described qualitative differences in fermentative performances. The release of compounds associated to the peaks at *m*/*z* 89.059, *m*/*z* 103.075, and *m*/*z* 117.093, tentatively identified as acetoin and esters, are coherent with claimed flavor properties of the investigated strains. We propose these mass peaks and their related fragments as biomarkers to optimize the aromatic performances of commercial preparations and for the rapid massive screening of yeast collections.

## 1. Introduction

Bread, one of the oldest fermented foods, is among the most popular and widely available staple foods in the world (especially in Western countries). Its nutritional and economic significance explains the remarkable efforts in research and development activities in the field of bread making. Bread flavor strongly influences consumer choice (with other sensory features such as aspect, taste, and texture) and is receiving increasing attention from the bakery industry [1]. Volatile organic compounds (VOCs), organic molecules generally characterized by molecular weight lower than 300 Dalton, and with considerable vapor pressure at room temperature [2], contribute to the global odor and flavor of bread [3]. Bread VOCs vary in quantity and quality as a function of ingredients, starter cultures, and process variables (and their interactions) [1,4,5,6]. Together with flour lipid oxidation and Maillard reactions, yeast fermentative activities can influence VOC release and diversity [4]. In the case of bread making, the most important protechnological microbe is the yeast *Saccharomyces cerevisiae*, responsible with its primary metabolic activity (transformation of fermentable carbohydrates to ethanol and carbon dioxide) of the alcoholic fermentation. Associated with the secondary metabolism of this organism is the release in the dough of a heterogeneous arena of volatile molecules (e.g., higher alcohols, aldehydes, sulfur-containing compounds, esters, phenols, carbonyl compounds, organic acids) which may affect bread sensory quality [7]. The diversity of *S. cerevisiae* strains and the quantity of yeast biomass mostly affect the formation of VOCs addressable to the fermentative activity of yeast [4,7]. Additionally, for this reason, the introduction and the use of bakery starter cultures is considered a keystone of current baking technology [8]. The fact that the quali-quantitative release of aroma-active compounds is a strain-dependent character within the species *S. cerevisiae* [1,7,9] leads to a noticeable interest in the selection and characterization of new yeast strains with specific aromatic potential [10]. The industrial interest is also testified by several patented solutions based on yeast strains [11]. In fact, since the year 2000, the number of patented yeast biotypes claiming aroma properties to answer the needs (of the baking industry) for consumer-oriented innovations in the fields [11] increased. Additionally, remaining in the sector of consumer-related economic relevance, it is interesting to note that the flavor ingredients represent a quarter of the total food additives market [12]. Moreover, the request to be informed on food and bread composition is arising among consumers and food companies in order to avoid synthetic additives [13] while looking for natural solutions to enhance sensory quality in fermented food [14].

Proton transfer reaction mass spectrometry (PTR-MS), belonging to the direct-injection mass spectrometric (DIMS) technologies [15], is an established method for the fast and direct online monitoring of volatile organic compounds in food without the need for any sample pre-treatment [15]. PTR-MS essentially relies on the chemical ionization of neutral compounds (M) [16,17] according to the following proton transfer reaction:

H_3_O^+^ + M → H_2_O + MH^+^ (1)

PTR-MS analysis is very rapid and can reach detection limits as low as 0.1 parts per trillion by volume [18,19,20,21,22,23]. The coupling of PTR-MS with Time-of-Flight (ToF) mass analyzers further improved time and mass resolution and allows the monitoring of VOCs during food fermentations [24,25,26,27]. While a previous study defined a PTR-MS-based protocol for simulating bread aroma during mastication [28], only recently PTR-ToF-MS was applied on bread fermentation through the online monitoring of volatiles during the leavening process and upon baking [5,6,9,29].

In contrast to other processes of industrial interest, such as beer, wine, and bioethanol production, where the performance of *S. cerevisiae* has been widely studied [30], the knowledge of the behavior of yeasts during solid-state processes, such as bread dough, is still limited. This observation is particularly pertinent if we refer to physiological aspects dealing with secondary metabolism, as in the case of the most part of microbial volatile organic compounds. In this work, we applied electronic microscopy for a preliminary characterization and PTR-ToF-MS analysis in order to characterize volatiles released by three commercial aromatic yeast strains during dough fermentation, aiming to (1) outline the possible molecular basis responsible of aroma-enhancing properties and (2) to individuate candidate VOCs suitable to be used as biomarkers for the rapid selection of new potential aromatic yeast strains.

## 2. Results

### 2.1. Commercial Aromatic Strains, SEM Visualization of Active Dry Yeasts, and Automated Monitoring of the Leavening Process

**Figure 1:**
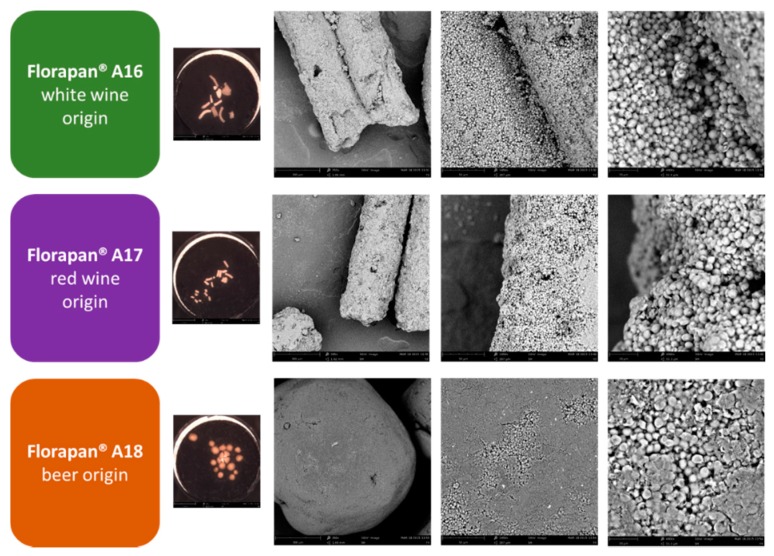
The SEM images of the active dry yeast surface for the commercial preparations Florapan^®^ A16 (A16), Florapan^®^ A17 (A17), and Florapan^®^ A18 (A18). Each preparation was visualized with an optical camera (~20X) (small colored image on the left) and with scanning electron microscope (SEM) (~250X, ~1500X, ~5000X) (grey images).

Volatile organic compounds were monitored during alcoholic fermentation occurring in three doughs respectively leavened by three *S. cerevisiae* biotypes selected and commercialized by reason of their aromatic properties in order to produce innovative products with new flavor attributes. Their use envisages a first phase with single inoculation of the starter in the dough preparation that suggested the separate addition of each active dry yeast to a dough and a fermentation for 16 h, in a sort of “levain”-like practice. The resulting dough can be used as starter for the preparation of bread, also in combination with a traditional commercial bakery starter culture. In our experimental design, giving the preliminary nature of our study and in order to clear understand the diversity of VOCs associated with the aromatic phenotype (avoiding possible synergistic/disruptive interactions with the traditional commercial bakery starter culture), we chose to follow the producer’s indications for the first phase. More in detail, and according to the manufacturer technical descriptions, the features of the analyzed *S. cerevisiae* strains were as follow: (1) Florapan^®^ A16 (A16) is a white wine aromatic yeast strain, characterized by a slow fermentation, that produces, in the dough, a clean and delicate bouquet; (2) Florapan^®^ A17 (A17) is a red wine aromatic strain with moderate fermentative performance and a fruity bouquet; and (3) Florapan^®^ A18 (A18) is an aromatic beer strain with moderate fermentative performance that leads to a round and complex flavor contribution (characterized by buttery and exotic notes) [31]. All three yeast preparations, traded in the form of dry active yeasts, were visualized by scanning electron microscope (SEM) (Figure 1).

Concerning the biomass preparation, it was already clear by microscopic analysis, a macroscopic difference in the aspect/kind of active dry yeasts produced to deliver A18 biomass, when compared with A16 and A17 biomasses (Figure 1). In all the cases, it was clear the presence of yeast cells presenting, in some cases, a sort of dehydrated phenotype (Figure 1).

In the analysis of volatile organic compounds, together with the “aromatic” experimental modes, we include, as a sort of control strain, a standard bakery strain (C), commercialized without any particular aromatic claimed properties. We prepared further “control” trials, in particular an empty vial (blank) and an un-inoculated dough. Preliminary trials on starter preparation had been necessary to assure that the used yeast biomass quantities led to the same yeast concentrations (CFU/mL) in the doughs for the four tested yeast strains. After the optimization of the fermentation conditions, we set up both the PTR-ToF-MS and the autosampler, to perform an automatic monitoring of the leavening process.

**Figure 2:**
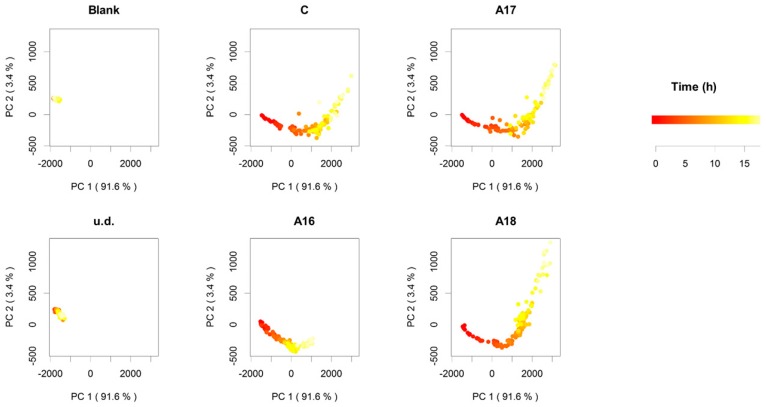
Volatile emission during leavening: principal component analysis of the autoscaled data for five intra-day repetitions for each experimental mode. Score plots for the first two principal components for blank, un-inoculated dough (u.d.), C, and for the three different yeasts, A16, A17, and A18, are depicted. Different color scale, from red to yellow, represents time evolution.

We analyzed the headspace in the vial every 62 min, generating up to 24 time points during the course of a typical leavening experiment. More than 460 peaks were detected during this leavening process. To simplify data analysis, we selected the principal volatiles by filtering the results on the basis of a 1 ppbV concentration threshold (5,9). The results obtained for the six experimental modes (blank, un-inoculated dough, C, A16, A17, and A18) were visualized by means of principal component analysis (PCA), with each point representing a distinct sampling point (Figure 2).

The result of blank (empty vial) and of un-inoculated dough confirmed that neither hexogen environmental VOCs nor volatiles connected with spontaneous fermentation in water/flour mixture may influence our observations (Figure 2). In addition, it was clear that the four commercial yeast preparations (C, A16, A17, and A18) were able to perform fermentation by reason of a clear evolution of the VOC profiles (Figure 2). Moreover, the distribution of variances in Figure 2 indicate possible differences in case of A16 (poor evolution) and A18 (pronounced evolution).

### 2.2. Ethanol Monitoring and Fermentative Performances

Among the selected subset of 63 mass peaks, we detected the parent ethanol peak (*m*/*z* 47.049) and two of the ethanol-related peaks (*m*/*z* 65.059 and 75.080) [32]. The ethanol monitoring had a dual significance in this experimental context: analytical and biotechnological. From an analytical point of view, it is essential to assess that the H_3_O^+^ (*m*/*z* 19) remained the primary ion in terms of signal intensity with regard to ethanol peaks (possible competing ionizer agent). Moreover, the massive presence of ethanol compromises the discrimination ability leading to complex mass spectra (because of peaks deriving from ethanol dimers and trimers, clusters between ethanol and water, and the corresponding fragments) [33]. From a biotechnological perspective, by observing the ethanol concentration, we have a direct analytic determination to evaluate yeast primary metabolic activity in the dough (during alcoholic fermentation sugars are fermented by yeast into CO_2_, ethanol, and other organic materials), while the standard bakery determination of leavening activity is indirectly determined based on the increase of dough volume (an indirect measurement of CO_2_ released by yeast in the dough). Considering that the parent ethanol peak (*m*/*z* 47.049) resulted saturated during our analysis, in order to assess ethanol production we followed the variation of two of the ethanol-related peaks (*m*/*z* 65.059 and 75.080) (Table 1).

Peak evolutions clearly indicated a comparable fermentative behavior for the strains C, A17, and A18, while a reduced fermentative activity had been detected in the cases of the strain A16. These findings were coherent with the technical data reported by the manufacturer for the three commercial aromatic strains, and in particular for the preparation A16 “characterized by a slow fermentation” [27].

### 2.3. Volatile Profiles and Aromatic Properties

Aiming to highlight the diversity of the yeast preparation in terms of released VOCs, we considered differences interesting to explain the claimed aromatic properties of these commercial bakery starter cultures. In this preliminary study, we focused on the dissimilarities detected for mass peak *m*/*z* 89.059, *m*/*z* 103.075, and *m*/*z* 117.093. In fact, we have to remember that volatile aroma-active esters formation, the largest and the most significant class of flavor compounds produced by fermenting yeast cells [34], is of particular interest in bread, because they are often characterized by having pleasant and fruity nuance [4]. Considering this chemical class and the analytical technique employed in this study, it must be taken into account that while, in some cases, esters can be monitored in the usual fashion through their protonated parent ions, it exists a consistent phenomenon of proton transfer-induced fragmentations [32]. In some cases, the fragmentation pattern solves the puzzle of tentative assignation, in other cases, the presence of fragments ensuing from a greater amount of esters, alcohols, sesquiterpenes, and monoterpenes did not permit adequate discrimination of carbonyl compounds. The signals observed at *m*/*z* 89.060 (sum formula: C_4_H_9_O_2_^+^; tentative identification: methyl-propanoate/ester fragment/acetoin) were considerably higher for A17 than for the standard commercial strain (Table 2). This peak, other than the primary ion for methyl-propanoate, is generally associated with major ester fragments, such as acetates, butanoates, isobutanoates, and pentanoates [32].

A similar trend has been reported for *m*/*z* 117.093 (sum formula: C_6_H_13_O_2_^+^; tentative identification: hexanoic acid/methylpropyl-acetate/ethyl esters) (Table 2), a peak commonly related to ethyl butanoate and ethyl isobutanoate. This evidence, together with the fragmentation pattern (reported as variation in abundance for mass peaks 39.022, 41.038, 43.956, 57.070, 61.028, 71.085, 89.059, 103.075, and 117.093 comparing VOC content at cycle 20 and at cycle 24; Table 3) analyzed in the light of the results reported by Aprea *et al.* [32], provide a concrete molecular basis to justify the fruity flavor claimed for A17 strain.

Considering the characteristics reported for the A18 strains, other than general increasing trends respecting to the C strain similar to those highlighted for A17 yeast preparation, we found two peaks markedly higher with respect to the other performant aromatic strain. In the first case, we remained in the field of esters as the variation involved the *m*/*z* 103.075 (sum formula: C_5_H_11_O_2_^+^; tentative identification 2-Methyl-butanoic acid/pentanoic acid/ethyl propionate), which is the protonated molecular ion [MH^+^] of ethyl propionate [35], a compound that, by reason of a pineapple-like odor, might be related to the exotic flavor ascribed to this commercial preparation. On the other hand, we detected a statistically significant augmentation for the *m*/*z* 89.059. In this case fragmentation (Table 3) information did not support the evidence that the detected increment is fully justified by a major tenor in esters. With this concern, it is useful to emphasize how *m*/*z* 89.059 represents the protonated molecular ion [MH^+^] of acetoin (23), a compound that, due to the buttery character, might be linked with the buttery notes claimed for the A18 bakery starter culture.

These preliminary data did not rule out the involvement of VOCs belonging to other molecular classes in the definition of aromatic properties claimed for these commercial preparations.

With concern of fragmentation patterns and methodological aspect, it is important to underline that, while PTR approach is usually characterized by a “soft” ionization conditions that minimize fragmentation phenomena, the presence of ethanol in the matrix negative influences these occurrences, reducing the analytical potential of the techniques. In previous works on commercial bakery starter cultures [5,9], we successfully used the methodology applied in this study that minimizes the ethanol effect (primary ion depletion and formation of ethanol clusters) by means of a sample dilution (ratio of 2:1) with an inert gas.

## 3. Discussion

Even though flavor perception is a consequence of the interaction of complex physicochemical, biochemical, physiological, psychological, and neurobiological phenomena [36,37,38], the study of volatile organic compounds associated with the headspace of a given food/beverage provides important information on the sensory quality of all kinds of edible matrices [39,40,41,42].

Microbial resources impact at different levels on the quality of cereal-base products [43,44,45,46,47], including the flavor features [1,4,5,6,11]. In particular, in light of the increasing attention towards “green” solutions to improve food quality, the use of aromatic-enhancing microorganisms offers the advantage to be a natural and sustainable solution that did not negatively influence the list of ingredients [14].

Considering the experimental design, we assured a common starting point in terms of CFU of yeasts per gram of dough to avoid differences addressable to the different amount of *S. cerevisiae* cells in the matrix. In fact, it has been lately proved that the initial level of yeast starter culture can influence the release of the majority of the volatile associated with the alcohol in the dough [1].

We proposed the monitoring of the two ethanol-related peaks *m*/*z* 65.059 and 75.080 as target VOCs to assess the progress of alcoholic fermentation in the dough. From one side, we reported findings suitable to describe the fermentative performances claimed by the manufacturer. On the other hand, even if we obtained similar trends for the analyzed mass peaks, they provided different descriptions of the intensity of the same phenomena, highlighting the need for further analysis before proposing this approach for quantitative analysis.

In the case of the dough prepared with the A16, the disposition of the points in the PCA (Figure 2) reflects the “slow fermentative behavior” claimed by the company technical sheet (behavior already confirmed by the trends of the ethanol-related peaks (Table 1). Oppositely, in the case of A18 strains we observe a variance distribution (Figure 2) susceptible to be linked to pronounced fermentation performances but, in this case, ethanol-related peak patterns do not confirm this remark (Table 1). In this last case, it is important to underline how, in *S. cerevisiae*, metabolic pathways responsible for volatile compound production are often part of the secondary cellular metabolism [48], hence, not necessarily released in a given proportion with respect to primary metabolites (ethanol in this case). In other terms, the differences of the point disposition on PCA representation in case of A18 were probably dependent on specific VOC modulations in the complex network of biochemical pathways that belong to the secondary yeast cellular metabolism.

More generally, the diversity in fermentative behavior is consistent with evidence reported by Aslankoohi *et al.* [30] that, studying the yeast fermentative aptitudes in the dough evaluating CO_2_ production, separated 19 moderate fermentative strains and five strains with a slow fermentative character, during the screening of 24 *S. cerevisiae* strains with dissimilar genetic backgrounds and belonging to different industrial applications (beer, wine, bioethanol, and bakery).

All of our findings connote the “slowly fermentative” starter culture A16 as a fable producer of VOCs when compared with the other commercial preparations. This evidence is not true if we normalize the peak intensities to the fermentative performances (in our case the normalization of the mass peaks *m*/*z* 89.060, *m*/*z* 103.075, and *m*/*z* 117.093 intensities as a function of the average of the two ethanol-related peaks *m*/*z* 65.059 and 75.080) (Table 4).

The application of PTR-MS in the rapid characterization of VOCs released by commercial aromatic bakery yeasts during dough fermentation indicates mass peaks as interesting markers and suggest a possible role of acetoin and esters. Esters are formed by chemical or lipase-catalyzed enzymatic condensation of organic acids and alcohols and by other several biochemical routes in microbes [49,50]. In effect, their concentrations correlated linearly with the concentrations of the corresponding higher alcohols, indicating that the availability of the precursors is the main limiting factor for the production of esters [51]. On the contrary, in our trials, comparing *S. cerevisiae* in the same “nutritive arena”, we stress the evidence of a strain-dependent features influencing esters production in yeast, findings in accordance with a recent “volatome” study that compares indigenous and commercial strains [52]. Interestingly, it is a subject of interest from an evolution perspective, given that esters are suggested to belong to the domestication phenotype of *S. cerevisiae* [7], given that the regulation of the synthesis of these compounds in the evolved strain differs from that in the ancestral strains [51] (strains used in food fermentations generally release more esters than their wild counterparts). While significant progress has been made regarding the molecular basis of ester formations, their cellular significance remains poorly understood [34]. Considering strain origins, it is mandatory to remember the relevant role of yeast esters’ biosynthesis during wine and beer productions [50,51,53,54].

With concern to acetoin release, this feature has already been described in strains isolated from the wine environment, where a general low production of acetoin was found to be the more common pattern in *S. cerevisiae* [55]. The high frequency of the low production phenotype (whereas it is possible to isolate high-producer strains) corroborates the genetic evidence of a dominant trait in the *S. cerevisiae* species [56]. Acetoin, intimately involved in the maturity of beer making [57], together with 2,3-butanediol, serves as a biomarker for yeast strain differentiation by reason of performance [58]. This observation is probably connected to the NAD-dependent butanediol dehydrogenase (Bdh1p) from *Saccharomyces cerevisiae* able to reversibly transform acetoin to 2,3-butanediol [59].

Intriguingly, in some points we touched subjects of common interest for yeast biology and evolution and for the biotechnological exploitation of yeast resources in the field sector [57,60]. From an integrated perspective, exometabolomic data are of key importance to understand physiological roles and genetic and biochemical regulation of complex network of biochemical pathways responsible for secondary metabolites production, such as those responsible of several microbial volatile organic compounds release [61]. With this aim, for example, Rossouw *et al.* [48] coupled comparative transcriptomics and metabolomics analysis in a wine-like model. Our findings suggest that this could be a subject of interest in the bread dough environment, in order to open new possibilities to design enhanced yeast selection strategies for increased aroma contributions.

On the whole, our findings led us to propose *m*/*z* 89.060, *m*/*z* 103.075, and *m*/*z* 117.093 mass peaks and their principal fragmentation patterns as biomarkers to optimize the VOC aromatic performances of these commercial preparations and for the rapid massive screening of yeast collection.

Considering possible future perspectives, a complete analysis of the spectra will be of help in detecting VOCs belonging to other molecular classes that can be implied in the definition of aromatic properties claimed for these commercial preparations. Additionally, considering all the limitations highlighted in our data analysis, we underline the importance to evaluate the combination of this technique with the new fast-GC PTR-TOF-MS to improve the analytical potential of the approach [33], a solution that helps (1) in the separate elution of ethanol and other volatiles avoiding interferences, excessive fragmentations, and dilution with inert gas, and (2) in tentative identifications by means of chromatographic separation of isomers.

## 4. Materials and Methods

### 4.1. Sample Preparation

Four different types of bakery starter cultures (one standard commercial preparation and three aromatic bakery yeast starter cultures) were used to obtain different dough samples, prepared according to the procedure described in the manufacturer specifications. The preparation was carried out using a bread homemaker machine (Princess Household Appliances, Lainate, Italy). The dough samples were supplemented with one of the following commercial starter cultures preparations: C (Lesaffre, Parma, Italy), A16 (Lallemand, Toulose, France), A17 (Lallemand), and A18 (Lallemand). The dough was divided into 3.0 g pieces (stored in 22 mL vials). During preparation, the dough was kept at 4 °C in order to slow the fermentation process. Then, the samples were transferred at 30 °C for the whole duration of the experiment (approximately 16 h). Un-inoculated dough had been used as a blank. Five replicates of each dough sample were analyzed (resulting in a total of 32 vials) and the entire experiment was repeated three times in different days. To confirm the absence of residual VOCs, an empty vial was introduced in the oven during baking and later used as a blank control.

### 4.2. SEM

Microstructures of the preparation were observed by scanning electron microscopy (SEM, Phenom proX, Phenom-World B.V., Eindhoven, The Netherlands).

### 4.3. Yeast Count

One gram of the dough was suspended in 9 mL of sterile physiological saline (8.5 g NaCl, g/L). Serial decimal dilutions were prepared in sterile physiological saline and 0.1 mL samples of appropriate dilutions were spread on sterile YPG agar plates (20 g/L glucose, 20 g/L yeast extract, 10 g/L peptone, and 20 g agar, pH 5.5). The plates were incubated at 26 °C for 48 h before counting the yeast colony forming units (CFU).

### 4.4. PTR-ToF-MS Analysis

In order to measure the headspace of the dough, a commercial PTR-ToF-MS 8000 apparatus from Ionicon Analytik GmbH (Innsbruck, Austria), was used in its standard configuration (V mode). The ionization conditions in the drift tube were the following: 110 °C, 2.30 mbar, 550 V. This led to an E/N ratio of about 140 Townsend (1 Td = 10^−17^ V·cm^2^). The inlet line consisted of a PEEK capillary tube (internal diameter 0.40 mm) heated at 110 °C. The inlet flow was set at 120 sccm and 40 sccm for dough and bread measurements, respectively.

The automation used for the leavening experiments was the same as the one described in previous works by Makhoul *et al.* [9] using an autosampler (Gerstel, Mulheim am Main, Germany) especially adapted to PTR-MS analyses. At the beginning of the experiment, the robotic arm moved the sample from a cooling tray where it was kept at 4 °C, to the incubation tray, whose temperature was set at 30 °C. Vials were then moved to the temperature-controlled purging site, connected to the PTR-ToF-MS inlet, and where the headspace analysis took place for 30 s with an acquisition rate of one spectrum per second. After measurement, the vial was moved to the incubation tray and the cycle was repeated on the following sample. This allowed for scanning of the tray [32 samples] in approximately one hour (1 cycle = 54 min). During leavening, the scan was repeated 16 to 20 times, in order to monitor the fermentation process. Due to the presence of relevant amounts of ethanol (an average of 20 ppmv of ethanol) in the headspace of the samples during leavening, an inert gas dilution was applied in an inert gas to sample ratio of 2:1. This permitted to prevent primary ion depletion and formation of ethanol clusters which might affect the final quantification of volatiles [32].

### 4.5. Data Analysis

Dead time correction, internal calibration of mass spectral data, and peak extraction were performed according to a procedure described in the works by Cappellin *et al.* [62,63], using a modified Gaussian peak shape. Peak intensity in ppbV was estimated by the formula described in literature [64], using a constant value for the reaction rate constant coefficient [k = 2 × 10^−9^ cm^3^·s^−1^]. This introduces a systematic error for the absolute concentration of each compound that is in most cases below 30% and could be accounted for if the actual rate constant coefficient is available [65].

All data detected and recorded by the PTR-TOF-MS were processed and analyzed using MATLAB (MathWorks, Natick, MA, USA) and in-house developed scripts written in the R programming language (R Foundation for Statistical Computing, Vienna, Austria).

## Figures and Tables

**Table 1:** PpbV variation for the mass peak *m*/*z* 65.059 and *m*/*z* 75.080 corresponding to the cycles 10, 15, 20, and 24 (leavening process driven by *S. cerevisiae* bakery starter cultures C, A16, A17, and A18).

*m/z*	Cycle	C(ppbV)	A16(ppbV)	A17(ppbV)	A18(ppbV)
65.058	10	44 ± 6 ^b^	10 ± 1 ^a^	49 ± 5 ^b^	45 ± 4 ^b^
65.058	15	73 ± 9 ^b^	19 ± 2 ^a^	85 ± 13 ^b^	74 ± 8 ^b^
65.058	20	66 ± 13 ^b^	20 ± 4 ^a^	79 ± 31 ^b^	69 ± 12 ^b^
65.058	24	119 ± 12 ^b^	54 ± 6 ^a^	144 ± 7 ^c^	142 ± 10 ^c^
75.080	10	155 ± 29 ^b^	18 ± 2 ^a^	181 ± 29 ^b^	155 ± 19 ^b^
75.080	15	277 ± 50 ^b^	37 ± 6 ^a^	341 ± 63 ^b^	287 ± 33 ^b^
75.080	20	252 ± 65 ^b^	41 ± 9 ^a^	332 ± 166 ^b^	270 ± 62 ^b^
75.080	24	512 ± 60 ^b^	133 ± 25 ^a^	704 ± 92 ^c^	637 ± 55 ^b^

Letters next to values indicate statistical significance.

**Table 2:** Concentrations (in ppb_V_) for the mass peak *m*/*z* 89.059, *m*/*z* 103.075 and *m*/*z* 117.093 corresponding to the cycles 10, 15, 20, and 24 (leavening process driven by *S. cerevisiae* bakery starter cultures C, A16, A17, and A18).

*m/z*	Cycle	C(ppbV)	A16(ppbV)	A17(ppbV)	A18(ppbV)
89.060	10	173 ± 58 ^b^	52 ± 3 ^a^	157 ± 17 ^b^	270 ± 37 ^c^
89.060	15	314 ± 53 ^b^	91 ± 14 ^a^	478 ± 78 ^c^	644 ± 88 ^d^
89.060	20	396 ± 77 ^b^	108 ± 15 ^a^	625 ± 168 ^c^	947 ± 137 ^d^
89.060	24	661 ± 110 ^b^	225 ± 20 ^a^	1261 ± 102 ^c^	1855 ± 182 ^d^
103.075	10	1.7 ± 0.5 ^b^	0.9 ± 0.2 ^a^	1.8 ± 0.1 ^b^	2.8 ± 0.3 ^c^
103.075	15	2.2 ± 0.4 ^b^	1.5 ± 0.3 ^a^	3.0 ± 0.4 ^c^	3.7 ± 0.4 ^c^
103.075	20	2.4 ± 0.4 ^a, b^	1.6 ± 0.4 ^a^	3.0 ± 0.5 ^b^	4.3 ± 0.6 ^c^
103.075	24	3.2 ± 0.7 ^a^	3.0 ± 0.7 ^a^	5.1 ± 0.6 ^b^	6.7 ± 0.6 ^c^
117.093	10	2.1 ± 0.8 ^b^	0.9 ± 0.1 ^a^	2.7 ± 0.2 ^b, c^	3.1 ± 0.4 ^c^
117.093	15	3.0 ± 0.6 ^b^	1.4 ± 0.2 ^a^	6.0 ± 1.3 ^c^	4.9 ± 0.4 ^c^
117.093	20	3.3 ± 0.5 ^a^	1.6 ± 0.3 ^a^	6.1 ± 1.7 ^b^	6.1 ± 0.9 ^b^
117.093	24	4.9 ± 0.6 ^a^	3.2 ± 0.3 ^a^	11 ± 1 ^b^	11 ± 1 ^b^

Letters next to values indicate statistical significance.

**Table 3:** Variations in terms of ppbV for mass peaks associated with the major ester fragments related to fragmentations involving the mass peak *m*/*z* 89.060, *m*/*z* 103.075 and *m*/*z* 117.093. Variations were calculated for C, A16, A17, and A18 comparing the concentration detected at cycle 20 with the concentration reported at cycle 24. These mass peaks are the more representative fragments for fragmentations involving the mass peaks *m*/*z* 89.060, *m*/*z* 103.075, and *m*/*z* 117.093, in accordance with fragmentation patterns describe in quality and quantity by Aprea *et al.* [32]

*m/z*	C (ppbV)	A16 (ppbV)	A17 (ppbV)	A18 (ppbV)
39.022	184.6	216.0	209.1	78.8
41.038	98.8	109.3	113.8	66.4
43.956	0.4	0.2	0.6	0.9
57.070	340.6	213.9	58.8	417.9
61.028	30.1	190.3	334.1	49.2
71.085	255.5	123.5	344.1	319.4
89.059	264.7	117.4	635.8	908.1
103.075	0.8	1.4	2.1	2.4
117.093	1.6	1.6	5.2	5.1

**Table 4:** A16/C concentration ratio for the mass peaks *m*/*z* 89.059, *m*/*z* 103.075, and *m*/*z* 117.093 and corresponding to the cycles 10, 15, 20, and 24, before and after normalization.

*m/z*	Cycle	A16/C (before normalization)	A16/C (after normalization)
89.059	10	0.300	2.027
89.059	15	0.291	1.791
89.059	20	0.273	1.431
89.059	24	0.342	1.150
103.075	10	0.529	3.582
103.075	15	0.682	4.196
103.075	20	0.667	3.487
103.075	24	0.937	3.156
117.093	10	0.429	2.900
117.093	15	0.467	2.872
117.093	20	0.485	2.536
117.093	24	0.653	2.198

## Abbreviations

The following abbreviations are used in this manuscript:

CFU	colony forming unit
DIMS	direct-injection mass spectrometry
GC	gas chromatography
PCA	principal component analysis
PEEK	polyether ether ketone
PTR-MS	proton-transfer-reaction mass spectrometry
PTR-TOF-MS	proton-transfer-reaction time-of-flight mass spectrometry
SEM	scanning electron microscopy
VOC	volatile organic compound
